# The Relationship between Positive Youth Development and Depressive Symptoms among Chinese Early Adolescents: A Three-Year Cross-Lagged Analysis

**DOI:** 10.3390/ijerph17176404

**Published:** 2020-09-02

**Authors:** Xinli Chi, Xiaofeng Liu, Qiaomin Huang, Xiumin Cui, Li Lin

**Affiliations:** 1 College of Psychology, Shenzhen University, Shenzhen 518060, China; 2170159004@email.szu.edu.cn (X.L.); cuixiumin13@126.com (X.C.); 2 Center for Lifestyle and Mental Health, Shenzhen University, Shenzhen 518060, China; 1810151024@email.szu.edu.cn; 3 Hong Chuang Kindergarten, Yi Cheng Center, Longhua District, Shenzhen 518131, China; 4 Law School, Shenzhen University, Shenzhen 518060, China; 5 Department of Applied Social Sciences, The Hong Kong Polytechnic University, Hong Kong, China; jocelyn.lin@polyu.edu.hk

**Keywords:** positive youth development, depressive symptoms, Chinese adolescents, cross-lagged study

## Abstract

Based on the development assets theory and the scar model, the present study examined the relationship between positive youth development (PYD) and depressive symptoms among Chinese early adolescents using a three-year longitudinal study design. Data from three waves were collected from 1301 students (Mean age = 12.46, SD = 0.63 years and 51.2% boys at wave 1) across the junior high school period (Grades 7–9). All participants completed a questionnaire that included the Center for Epidemiologic Studies Depression Scale (CES-D) and the Chinese Positive Youth Development Scale (CPYD) once a year over three years. After controlling for age and gender, this study found that PYD significantly predicted subsequent depressive symptoms. However, depressive symptoms did not significantly predict subsequent PYD. The results indicated a unidirectional relationship between PYD and depressive symptoms, where a reduction in PYD may increase subsequent depressive symptoms, though not vice versa. Besides, the negative cross-sectional correlation between PYD and depressive symptoms remains significant and stable from first year (T1) to third year (T3). These findings suggest that promoting PYD may be a promising approach to preventing/reducing adolescent depressive symptoms.

## 1. Introduction

Depression is one of the most common mental disorders and a major public health problem, especially in adolescents [1,2]. For example, some studies found that an estimated 8–20% of adolescents have depressive symptoms globally [3,4]. A recent meta-analysis study found that the prevalence of depressive symptoms among adolescents in China was about 24.3% [2]. Shenzhen, which is located in the south-east of China, is one of China’s fastest-growing cities and often dubbed “China’s Silicon Valley”, with a Gross Domestic Product (GDP) ranking third (the first and second are Shanghai and Beijing) in China in 2018 [5]. Research has documented that life is fast and students are under great academic competition and pressure in economically developed places. These factors may lead to higher risks of adolescents experiencing depressive symptoms [6]. Consistent with this, a recent study found that the prevalence of depressive symptoms among Grade 7 and Grade 9 students in Shenzhen was 33.4% and 28.8%, respectively [7], which was relatively higher than what have been found in other cities of China [2]. Therefore, it is necessary to pay special attention to adolescents in economically developed cities like Shenzhen. A large number of studies have found that depressive symptoms are associated with a series of negative outcomes, including academic performance failure [8], interpersonal relationship conflicts, anxiety and insomnia [9]. Moreover, the duration and continuity of depressive symptoms also increases the risk of self-harm and even suicidal behaviors [10,11], and these negative effects may continue into adulthood [12]. Thus, it is particularly urgent to examine potential factors protecting against depressive symptoms among adolescents, especially from economically developed city such as Shenzhen. This study is expected to help provide some theoretical and practical implications for the design of prevention or intervention strategies to reduce adolescent depression.

In recent years, many studies have examined the association of positive psychological qualities with depressive symptoms among adolescents [13,14,15]. However, it remains unclear whether possessing insufficient positive psychological qualities leads to the occurrence of depressive symptoms, or whether depressive symptoms interfere with the development of positive attributes among adolescents. Two models may help explain the relationship between positive psychological features and depressive symptoms. The development assets theory suggests that the occurrence of internalized and externalized problems may be due to a lack of developmental assets, such as positive psychological resources (e.g., resilience and self-efficacy) [16,17]. The fewer assets adolescents have, the more likely they are to have internalizing and/or externalizing problems, and the less likely they are to develop healthily [18].

Essential to development assets theory is the positive youth development (PYD) perspective. The PYD perspective calls for a shift from incapacities and deficits to strengths and potentials in the conceptualization of adolescence [19]. It contends that adolescents should be treated as “resources to be developed” rather than “problems to be solved” [20]. However, the views about what comprise PYD are not consistent. Generally, PYD refers to the potential of individuals, in terms of talents, strengths, interests, and so on, instead of lack of ability [19]. Lerner operationalized PYD has five positive attributes—competence (e.g., social competence and cognitive competence), confidence (e.g., self-efficacy), connection (i.e., relatedness with significant ones), care (e.g., sympathy and empathy) and character (e.g., internal standards for right behavior) [21]. Benson regarded PYD as 40 developmental assets, of which 20 are internal assets (e.g., commitment to learning, social competence and positive identity) and another 20 are external assets (e.g., empowerment, support, and boundaries and expectations) [22]. Catalano et al. proposed 15 attributes of PYD, specifically bonding, self-determination, resilience, self-efficacy, cognitive competence, social competence, emotional competence, behavioral competence, moral competence, spirituality, clear and positive identity, belief in the future, recognition of positive behavior, prosocial involvement, and prosocial norms [23]. Despite the incongruence in the operationalization of PYD, scholars holding the PYD perspective generally believe that adolescents equipped with PYD attributes are more likely to live a thriving life marked by higher levels of positive functioning and lower levels of risks and problems [19,21,22]. These positive attributes probably enable adolescents to handle their personal problems in a healthy way, and prevent them from the harm of risk factors imposed by environment.

Previous studies have consistently shown that PYD was strongly correlated with depressive symptoms among adolescents [13,24,25]. For example, a cross-sectional study found that lower levels of PYD features were associated with a greater prevalence of depressive symptoms among adolescents in the northeast United States [26]. Recent studies based on the Chinese context also reported consistent findings [7,27]. Although previous studies examined the correlation between PYD and adolescent depressive symptoms, studies based on longitudinal data are insufficient, especially in the mainland Chinese context. Among the few longitudinal studies conducted, a study based on three waves of longitudinal data found that PYD in Grade 5 predicted lower levels of depressive symptoms in Grade 6 [28]. Another study found that PYD could significantly predict low levels of depressive symptoms over time in Hong Kong adolescents [29]. A more recent study found that PYD attributes in Grade 7 students negatively predicted their depressive symptoms in Grade 8, and depressive changes over time [30]. Thus, based on the development assets theory and previous research, the present study hypothesized through a longitudinal design that PYD would predict depressive symptoms among Chinese junior high school students.

Another model, the scar model, may explain the predicting effect of depressive symptoms on PYD features among adolescents [31]. It proposes that the experience of having suffered from depression leaves “scars” in individuals that have a lasting effect on psychological resources, such as self-esteem, self-efficacy and resilience. The model suggests that a decrease in positive attributes is a consequence of experiencing mental health problems, such as depressive symptoms [32]. Previous studies have demonstrated that depressive symptoms could affect an adolescent’s cognitive, affective and behavioral functioning development and could lead to low psychological resources, such as self-esteem, resilience, and social competence [33,34,35]. For example, cross-sectional studies found that depressed adolescents, compared to nondepressed adolescents, have poorer social skills, negative bonding with others, and a low level of subjective well-being [36,37]. Thus, based on the scar model and previous studies, the present study speculated that depressive symptoms would reduce the level of PYD over time among Chinese adolescents.

**Figure 1:**
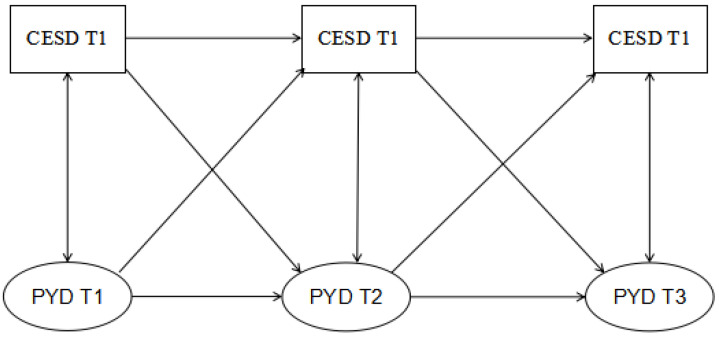
The hypothesized full longitudinal cross-lagged model. Cross-lagged relationship between positive youth development (PYD) and depressive symptoms across three waves; T1, Time 1 (first year); T2, Time 2 (second year), T3, Time 3 (third year).

Against the developmental assets theory, the scar model and previous research, the present study examined four competing hypotheses regarding the direction of effects between PYD and depressive symptoms, as follows: (1) PYD and depressive symptoms did not influence each other directly, but shared a variance caused by unmeasured factors (i.e., stability model); (2) PYD had a direct effect on depressive symptoms; (3) Depressive symptoms had a direct effect on PYD; and (4) PYD and depressive symptoms demonstrated reciprocal effects. The hypothesis models were expected to deepen the understanding of the causes and effects of depressive symptoms in adolescents. The findings were expected to help develop targeted approaches to prevent/reduce adolescent depressive symptoms (Figure 1).

## 2. Methods

### 2.1. Participants

The present study was based on three waves of data collected during three junior adolescent years in Shenzhen, Guangdong Province, China. There are nine districts in Shenzhen (e.g., Luohu, Futian and Nanshan), from which six districts were randomly selected. In these six districts, a school in each district was selected as our research school. Students in these 6 schools were invited to be our research participants. Further, the eligible participants were selected with the following criteria: (1) adolescents who were in Grade 7, (2) adolescents who received consent to participate from their guardians, and (3) adolescents who agreed to participate. A data sample at baseline (Time 1, n = 1544) was collected in October 2016. Follow-up data were collected one year later (Time 2, n = 1511), when students were in Grade 8, and two years later (Time 3, n = 1480), when they were in Grade 9. Among the three waves, some adolescents did not participate in Grades 8 and 9. This could be because some participants moved to other schools or were absent from their class. Therefore, 1301 adolescents participated in three surveys as the final sample, which included 666 males and 621 females, and 14 students did not report their gender. The mean age of participants was 12.46 years (SD = 0.63) at wave 1.

### 2.2. Procedure

Participants were invited to attend a 20 min test in the form of paper-and-pencil questionnaires in a classroom setting. Before the tests, graduate students in psychology and school youth counselors were trained to supervise the tests. They introduced the study purposes and provided prompt instruction to participants during the tests. The participants completed the questionnaires voluntarily and anonymously in the absence of their teachers. Data confidentiality was also guaranteed. Across three time points, the same self-completed questionnaires were distributed to all participants on-site in the classroom setting. No compensation was provided. Written informed consent was obtained from all participants of the study and their parents. The research protocol was approved by the administration committees of the surveyed schools and the Human Research Ethics Committee of Shenzhen University (NO. 20160516, approved on 22 June 2016).

### 2.3. Measurements

#### 2.3.1. Chinese Positive Youth Development Scale

To examine PYD in Chinese adolescents, the Chinese Positive Youth Development Scale (CPYD) was selected [38,39]. The CPYD divided 15 traits proposed by Catalano et al. into four high-order dimensions: cognitive-behavioral competence, positive identity, prosocial attributes and general PYD qualities. The cognitive-behavioral competence subscale included nine items from the cognitive competence, self-determination and behavioral competence dimensions, which measure each respondent’s perceived cognitive and behavioral skills. The positive identity subscale included six items related to beliefs about the future, and clear and positive identity dimensions, which measured the skills of developing a healthy identity and establishing future goals. The prosocial attributes subscale included six items from the prosocial involvement and prosocial norms dimensions, which measure the tendency to internalize and adopt the norms of social responsibility and to engage in behaviors that benefit other people. The general PYD qualities subscale included 23 items from the resilience, social competence, self-efficacy, moral competence, bonding, recognition of positive behavior, spirituality and emotional competence dimensions. The subscale measured the rest of the PYD constructs. Each item was rated on a 6-point Likert scale (1 = strongly disagree, 6 = strongly agree). An average score indicates adolescents’ PYD, with higher scores reflecting a higher tendency among adolescents to have more positive psychological qualities. In previous studies, the CPYD exhibited good reliability and validity [40,41]. In this study, the CPYD showed acceptable reliability and validity (see Table 1).

#### 2.3.2. Center for Epidemiologic Studies Depression Scale

The Chinese version of the 20-item Center for Epidemiological Studies Depression Scale (CES-D) was used to assess the frequency of depressive symptoms in the last week [42,43]. Sample items on this scale include “I felt fearful” and “My sleep was restless.” Each participant was asked to rate the items on a 4-point Likert scale with responses ranging from “0 = *rarely or none of the time*” to “3 = *almost or all of the time*.” A higher total score in the CES-D indicates a higher level of depressive symptoms. The Chinese version of the scale indicated high internal consistency for use with adolescents in previous research [44]. CES-D had satisfactory reliability and validity in the present study (see Table 1).

### 2.4. Statistical Analysis

After entering the data into the computer, replacement values for missing data were estimated using the Expectation-Maximization (EM) algorithm to maximize statistical power [45]. Then, SPSS 22.0 was used for partial analysis to examine the correlations between the four second-order PYD constructs and depressive symptoms. Latent variable structural equation modeling (SEM) with MPLUS 8.0 was employed to test the autoregressive cross-lagged longitudinal model. The autoregressive cross-lagged model is an analysis method utilized to examine the predicting effect of one variable in the past on the variable in the future, as well as the reciprocal links or directional impacts between two or more variables over time. When individuals enter puberty, the depressive symptoms levels of girls are significantly higher than those of boys [46]. Additionally, previous studies have found that the depressive symptoms of adolescent change with age [47]. Therefore, gender and age were controlled when conducting the model in this study. To examine the relationship between PYD and depressive symptoms, five models were constructed. First, the baseline model only included the autoregressive regression of PYD and depressive symptoms, which did not include the cross-lagged path. Second, the two unidirectional models of the relationship between PYD and depressive symptoms were constructed, respectively. Third, the bidirectional model and the modified model of the relationship between PYD and depressive symptoms were constructed. Goodness of fit was assessed with the following fit indices: comparative fit index (CFI), Tucker–Lewis index (TLI), root mean square error of approximation (RMSEA) and standardized root mean square residual (SRMR). The following thresholds were used: for CFI and TLI excellent fit > 0.95 and moderate fit > 0.90; for RMSEA and SRMR excellent fit < 0.05 and moderate fit < 0.08. Meanwhile, Cronbach’s alpha (Cronbach’s α), McDonald’s omega (McDonald’s ω), omega and the average variance extracted (avevar) were examined for the reliability of CPYD and CES-D.

## 3. Results

### 3.1. Descriptions of PYD Correlates and Depressive Symptoms between Retained and Dropped Participants

As can be seen in Table 2, among participants measured at baseline, 15.7% (*n* = 243) could not be matched in the third survey. There were significant differences in age and depressive symptoms scores, as well as cognitive-behavioral competencies (CBC) between the retained and dropped participants. However, gender, positive identity (PI), prosocial attributes (PA) and general positive youth development qualities (GPYDQ) were not significantly different between the two groups.

### 3.2. The Development Trend of Depressive Symptoms and PYD

As shown in Table 3, a repeated-measures multivariate analysis of variance (MANOVA) was conducted with the time (T1, T2 and T3). For CESD, a significant effect of time was observed, *F* (1.94, 2525.51) = 12.53, *p* < 0.001, η^2^ = 0.01. Pairwise comparisons showed that the difference between T1 and T2 was not significant (*p* > 0.05, *d* = −0.01), but the CESD scores at T1 were higher than the scores at T3 (*p* < 0.001, *d* = 0.14), and the CESD scores at T2 were lower than the scores at T3 (*p* < 0.001, *d* = 0.15).

For cognitive-behavioral competence, a significant effect of time was observed, *F* (1.95, 2541.55) = 66.07, *p* < 0.001, η^2^ = 0.05. Pairwise comparisons showed that the difference between T1 and T2 was not significant (*p* > 0.05, *d* = 0.03), but the cognitive-behavioral competence scores at T1 were lower than the scores at T3 (*p* < 0.001, *d* = 0.32), and the CESD scores at T2 were lower than the scores at T3 (*p* < 0.001, *d* = 0.34).

For positive identity, a significant effect of time was observed, *F* (1.95, 2528.48) = 20.00, *p* < 0.001, η^2^ = 0.02. Pairwise comparisons showed that scores at T1 were higher than scores at T2 (*p* < 0.05, *d* = 0.08) and lower than the scores at T3 (*p* < 0.001, *d* = 0.11), and the positive identity scores at T2 were lower than those at T3 (*p* < 0.001, *d* = 0.19).

For prosocial attributes, a significant effect of time was observed, *F* (2, 2600) = 26.91, *p* < 0.001, η^2^ = 0.02. Pairwise comparisons showed that the difference between T1 and T2 was not significant (*p* > 0.05, *d* = 0), but the prosocial attributes scores at T1 were lower than the scores at T3 (*p* < 0.001, *d* = 0.21), and the prosocial attributes scores at T2 were lower than the scores at T3 (*p* < 0.001, *d* = 0.21).

For general PYD qualities, a significant effect of time was observed, *F* (1.93, 2517.05) = 70.05, *p* < 0.001, η^2^ = 0.05. Further analyses indicated that the general PYD qualities increased year by year. The difference of general PYD qualities between T1 and T2 was not significant (*p* > 0.05, *d* = 0.01), but the scores at T1 were lower than the scores at T3 (*p* < 0.001, *d* = 0.34), and scores at T2 were lower than the scores at T3 (*p* < 0.001, *d* = 0.32).

### 3.3. Correlations between the Four Second-Order PYD Constructs and Depressive Symptoms

After controlling for gender and age, the correlations between the subscales of PYD and depressive symptoms are shown in Table 4. The relationships of cognitive-behavioral competence, positive identity, prosocial attributes and general PYD qualities with depressive symptoms were significantly negatively correlated at all three time points. The findings indicated that the level of depressive symptoms decreased as the levels of the four second-order PYD constructs increased, and vice versa.

### 3.4. Cross-Lagged Regression Analysis

Based on a correlation analysis, the four second-order PYD constructs and depressive symptoms were entered into the constructed autoregressive cross-lagged model after controlling for age and gender. MPLUS 8.0 was used to calculate the model fit index and standardized regression coefficients using maximum likelihood estimation for the model.

**Figure 2:**
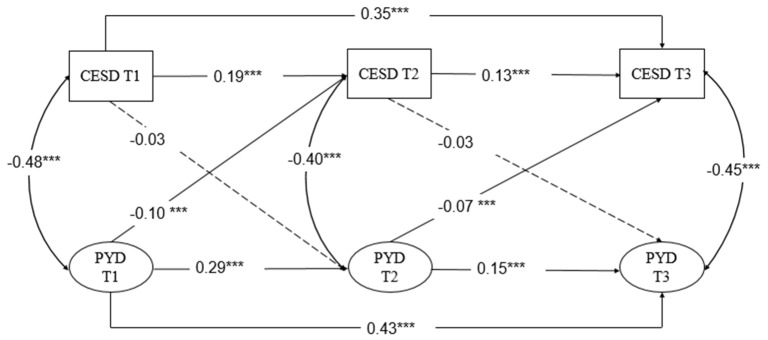
Cross-lagged relationship between positive youth development (PYD) and depressive symptoms across three waves. T1, Time 1 (first year); T2, Time 2 (second year); T3, Time 3 (third year). *Note.* *** *p* < 0.001.

The results are shown in Table 5. Compared to the baseline model, both the unidirectional models and the bidirectional model had better fit indices. Furthermore, the fit indices of the bidirectional model were better than the two unidirectional models. Thus, the bidirectional model was used to examine the relationship between PYD and depressive symptoms. In addition, to control the development stability of the same variable, an autoregressive path was allowed between the same variables at two time points. Therefore, based on the bidirectional model, the second-order autoregressive path was added according to the modified index in this study, and the final modified model was obtained. The two added paths included were from T1 PYD to T3 PYD (*β* = 0.40, *p* < 0.001), and from T1 depressive symptoms to T3 depressive symptoms (*β* = 0.35, *p* < 0.001). As seen in Table 4, the path analyses revealed that PYD and depressive symptoms are significantly correlated with one another from T1 to T3. Compared to the bidirectional model, the modified model showed better fit indices, as follows: *χ*^2^ = 821.20, *df* = 98, CFI = 0.95, SRMR = 0.04, RMSEA = 0.08, 90%CI = [0.07, 0.08]. As shown in Figure 2, all paths from PYD to depressive symptoms were significant (T1 → T2: *β* = −0.10, *p* < 0.01; T2 → T3: *β* = −0.07, *p* < 0.05), while all paths from depressive symptoms to PYD were not significant (T1 → T2: *β* = −0.03, *p* > 0.1; *T2* → T3: *β* = −0.03, *p* > 0.1). These findings indicate a unidirectional negative relationship between PYD and depressive symptoms; specifically, PYD may result in depressive symptoms.

## 4. Discussion

The present study found that depression symptoms in Chinese adolescents were stable in Grade 7 and Grade 8, and slightly declined in Grade 9. This result was consistent with previous research conducted among adolescents in Beijing [47]. Students at Grade 7 faced many of the pressures and challenges of adapting to the new environment when they were in the transition from primary school to middle school. After entering the 8th grade, these adolescents have to face new self-development tasks. At this stage, they often experience rebelliousness, self-centeredness, confusion between reality and illusion, and confusion of self-identity [48]. These factors may lead to depressive symptoms in adolescents in the first two years during the junior middle school period. After Grade 9, most adolescents would focus more on their learning, and may try to adjust their emotions be ready for high school entrance examinations. In addition, in Grade 9, parents and teachers give more support and understanding to students, reducing the negative effects of negative emotions on academics. Therefore, the depression symptoms of teenagers in Grade 9 were alleviated compared with the first two years.

Regarding features of PYD, while cognitive-behavioral competencies, general positive youth development qualities and prosocial attributes are stable in Grade 7 and Grade 8, and slightly increased in Grade 9, positive identity slightly declines from Grade 7 to Grade 8 and increases in Grade 9. There are two points worth noting. Firstly, generally speaking, PYD features, except for positive identity, increase slightly with grades, which is related to the gradual maturity of adolescents’ own physical and mental development and the gradual adaptation to the school environment and learning content. Secondly, the level of positive identity first declines and then rises. According to Erickson’s theory of social psychology development [49], the important developmental task of adolescence is gaining a coherent identity and avoiding identity. Adolescents aged 11–13 years (almost at Grade 7 and 8) are in a time of disorganization because of physical and psychological development [50]. Individuals at this stage have to face new school environments (middle school), new roles (middle school students), and new learning content. These issues may thereby make the positive identity of students decrease from Grade 7 to 8.

Regarding the cross-lagged analysis results, the study showed that PYD significantly predicted subsequent depressive symptoms, indicating that poor PYD leads to depressive symptoms. However, depressive symptoms did not significantly predict subsequent PYD. In other words, there was unidirectional influence between PYD and depressive symptoms. The result supported the development assets theory that lacking PYD could lead to adolescent depressive symptoms. These findings were similar to previous studies showing that several or multiple constructs of PYD significantly predict problematic adolescent behaviors, including depressive symptoms [28,35,51,52]. Adolescents with a high level of PYD may have an optimistic and positive attitude, which could help them actively cope with negative events or adverse situations, thereby reducing the negative impact on their own mental health [53,54,55]. Meanwhile, as a collective term for important multiple psychological resources, PYD could encourage individuals to develop corresponding positive psychological resources (i.e., cognitive ability, emotional regulation ability) in response to negative events [56,57,58]. The findings support the major proposition of the PYD perspective: having strengths prevents adolescents from developing poor mental health, such as depressive symptoms [59]. The findings may also contribute to the development of positive psychology in non-Western contexts, and imply that cultivating positive characteristics/strengths among Chinese adolescents may be a future direction of intervention.

Of note, depressive symptoms could not significantly predict PYD, which was inconsistent with previous studies. Previous research showed that depressive symptoms could negatively affect the psychosocial competences of adolescents [33,60]. For example, researchers found that depressive symptoms could damage the cognitive function system of an individual, and could lead to impaired psychosocial abilities [61,62]. In addition, several studies found that adolescents with high levels of depressive symptoms had lower levels of resilience [63,64]. There were several plausible explanations for the inconsistent findings in this study with previous studies. The one reason may be that the present study used PYD from an integrated perspective rather than a single dimension. Adolescent depressive symptoms may lead to low levels of one or several aspects of PYD, and may not impact other features of PYD [63,64]. Another reason may be that depressive symptoms, such as mild to moderate depressive symptoms, in some adolescents constitute a transient developmental phenomena that will naturally disappear as they grow older, when physical and mental development gradually matures [2,65,66]. This point was further supported by the current findings showing that depressive symptoms were stable in Grade 7 and 8, and declined in Grade 9. In this situation, PYD may not have been significantly affected. Third, it is also possible that the causal relationship between PYD and depressive symptoms is moderated by other factors, such as social support. Previous studies found that among depressed adolescents, those who reported greater perceived social support from parents, friends and classmates tended to display a higher level of psychosocial functioning than did their counterparts who reported lower social support [67,68]. The current findings did not support scar theory, so it is better to articulate how the current findings help with refining scar theory. Obviously, more in-depth studies, such as those that employ quantitative methods including more waves of longitudinal data, and qualitative methods such as focus group interviews or face-to-face in-depth interviews, are needed so as to explore the potential effects of different moderators and to further examine the reciprocal model in the future. Overall, a low level of PYD or a lack of PYD resulted in adolescent depressive symptoms, while depressive symptoms did not lead to a decrease in PYD. The study indicated that depressive symptoms may be the outcome, and PYD occurs before the development of depressive symptoms among junior high school students in modern cities of China. The study highlights the importance of promoting PYD characteristics among adolescents for preventing/intervening in depressive symptoms.

Moreover, this study finds that the cross-sectional correlation between PYD and depressive symptoms remained stable and significant from T1 to T3 in a cross-lagged SEM with latent variables. These results are consistent with the results of Pearson’s correlation. After controlling for initial PYD and depressive symptoms, the relation of two variables can accurately illustrate the change in the cross-sectional association of PYD with depressive symptoms. The results further indicate that the relationship between PYD and depressive symptoms remained stable and significant over time. The findings indicated that during a three-year junior high school period, PYD and depressive symptoms share many common predictive factors; thus, the utility of the correlation between them was strong and remained stable. This suggested further that it would be a promising approach to promote PYD in the fight against depressive symptoms among Chinese junior high school students.

This study had some limitations. First, adolescent depression symptoms were only examined at three time points across three school years. To explore the long-term developmental patterns of depressive symptoms in adolescence, more data waves over a longer period, such as the six years from Grade 7 to Grade 12, could be included. Second, the present study used the self-reporting approach. Adolescents may under-report their depressive symptoms because of social desirability. Future studies could include other report approaches (e.g., parent, teacher). Third, although the study determined that PYD could affect adolescent depressive symptoms, the underlying relationship between four second-order PYD constructs (i.e., cognitive-behavioral competence, prosocial attributes, positive identity and general PYD qualities) and adolescent depressive symptoms is unknown. Future studies could explore the specific relationships of the four second-order PYD constructs with adolescent depressive symptoms, respectively. Finally, as the current study was based on a sample of Shenzhen junior students, it is not a given that the findings hold in other cities in China.

Despite these limitations, the study found that a low level of PYD could result in depressive symptoms, while depressive symptoms did not reduce the level of PYD. The findings have some important implications for managing adolescent depressive symptoms. Educators and family members may focus on helping adolescents increase their PYD traits (e.g., resilience, self-efficacy and self-esteem) to prevent/reduce depressive symptoms. Previous studies revealed that PYD could be increased through training, such as Positive Adolescent Training through Holistic Social Programs [69]. Educators may implement courses and activities that aim to improve PYD. As for family, programs such as Sport-Based Life Skills Programs could help improve adolescents’ PYD within the family context through physical activities [70]. In this way, the negative impacts of depressive symptoms may be reduced or eliminated.

In short, although internet addiction among Hong Kong adolescents tended to decline over a period of 4 years, the overall prevalence rate remained high. While economic disadvantage and family non-intactness were risk factors, family functioning and positive youth development were protective factors in the development of adolescent internet addiction. Based on the present findings, it is suggested that more attention from the public and academia should be given to the phenomenon of internet addiction in Hong Kong, and that the promotion of family functioning and positive youth development attributes in adolescents could be promising actions in preventing adolescent internet addiction.

## 5. Conclusions

In short, this study found that PYD significantly predicted subsequent depressive symptoms. However, depressive symptoms did not significantly predict subsequent PYD. The results indicated a unidirectional relationship between PYD and depressive symptoms, where a reduction in PYD may increase subsequent depressive symptoms, though not vice versa. Additionally, the negative cross-sectional correlation between PYD and depressive symptoms remains significant and stable over time. Based on the present results, it is suggested that the promotion of positive youth development attributes in Chinese adolescents could be a promising action in preventing adolescent depressive symptoms.

## Figures and Tables

**Table 1:** Psychometric characteristics of CPYD and CES-D.

Scales	*χ* ^2^	*df*	CFI	TFI	RMSEA	SRMR	McDonald’s ω	Cronbach’s α	Omega	Avevar
*CPYD*										
CBC-T1	4141.522	36	0.992	0.975	0.046	0.015	0.862	0.861	0.862	0.412
CBC-T2	4582.095	36	0.997	0.989	0.032	0.012	0.872	0.871	0.872	0.433
CBC-T3	8326.689	36	0.995	0.986	0.049	0.010	0.933	0.932	0.933	0.607
PA-T1	1882.882	10	0.999	0.995	0.026	0.007	0.802	0.796	0.800	0.450
PA-T2	2110.620	15	0.998	0.993	0.028	0.007	0.785	0.780	0.780	0.420
PA-T3	5441.852	15	0.996	0.978	0.078	0.014	0.908	0.906	0.906	0.300
CB-T1a	3612.898	15	0.995	0.983	0.056	0.000	0.872	0.870	0.872	0.537
CB-T2	3872.282	15	0.992	0.970	0.077	0.014	0.882	0.881	0.882	0.558
CB-T3	6005.443	15	0.999	0.997	0.030	0.004	0.926	0.922	0.924	0.672
GPYDQ-T1	10,425.744	190	0.933	0.917	0.059	0.042	0.914	0.913	0.913	0.346
GPYDQ-T2	11,772.895	190	0.935	0.916	0.063	0.043	0.922	0.921	0.921	0.370
GPYDQ-T3	19,814.318	190	0.968	0.959	0.057	0.029	0.958	0.957	0.957	0.530
*CES-D*										
CES-D-T1	8463.780	190	0.930	0.905	0.056	0.040	0.874	0.849	0.843	0.236
CES-D-T2	8817.822	190	0.959	0.0942	0.045	0.029	0.878	0.853	0.847	0.243
CES-D-T3	12,802.648	190	0.957	0.944	0.053	0.033	0.909	0.881	0.873	0.283

*Note*. T1, Time 1 (first year); T2, Time 2 (second year), T3, Time 3 (third year). CPYD = Chinese Positive Youth Development Scale; CES-D = Center for Epidemiologic Studies Depression Scale; CBC = cognitive-behavioral competencies; GPYDQ = general positive youth development qualities; PI = positive identity; PA = prosocial attributes.

**Table 2:** Comparisons of PYD correlates and depressive symptoms of the participants measured at baseline by loss to follow-up.

Variable	Follow-up (*n* = 1301) *n*/*M*	%/SD	Loss to Follow-up (*n* = 243) *n*/*M*	%/SD	*p*
Gender					0.12
Male	666	51.2	193	57.2	
Female	621	47.5	104	42.8	
Missing data	14	1.1	_	_	
Age					0.01
11	23	1.8	1	0.4	
12	719	55.3	119	49.0	
13	487	37.4	108	44.4	
14	60	4.6	11	4.5	
15	4	0.3	3	1.2	
Missing data	8	0.6	1	0.4	
CES-D	13.66	9.15	15.35	9.07	0.01
CBC	4.77	0.78	3.61	0.58	0.00
PI	4.82	0.94	4.71	0.93	0.08
PA	4.11	0.74	4.06	0.74	0.35
GPYDQ	4.85	0.71	4.84	0.69	0.76

**Table 3:** Repeated measures related to depressive symptoms and PYD.

	CES-D *M* ± *SD*	CBC *M* ± *SD*	PI *M* ± *SD*	PA *M* ± *SD*	GPYDQ *M* ± *SD*
T1	13.66 ± 9.15	4.77 ± 0.78	4.82 ± 0.94	4.93 ± 0.88	4.85 ± 0.71
T2	13.76 ± 9.32	4.75 ± 0.81	4.74 ± 0.98	4.93 ± 0.82	4.86 ± 0.74
T3	12.40 ± 9.31	5.03 ± 0.83	4.93 ± 0.98	5.11 ± 0.87	5.10 ± 0.77
F	12.53 ***	66.07 ***	20.03 ***	26.91 ***	70.05 ***
η^2^	0.01	0.05	0.01	0.02	0.05

*Note*. T1, Time 1 (first year); T2, Time 2 (second year); T3, Time 3 (third year). CESD = Center for Epidemiologic Studies Depression Scale; CBC = cognitive-behavioral competencies; GPYDQ = general positive youth development qualities; PI = positive identity; PA = prosocial attributes. *** *p* < 0.001.

**Table 4:** Correlations of the four second-order PYD constructs with depressive symptoms.

	1	2	3	4	5	6	7	8	9	10	11	12	13	14	15
1. CES-D-T1	1.00	-													
2. CES-D-T2	0.25 ***	1.00	-												
3. CES-D-T3	0.42 ***	0.25 ***	1.00	-											
4. CBC-T1	−0.33 ***	−0.15 ***	−0.22 ***	1.00	-										
5. PI-T1	−0.45 ***	−0.19 ***	−0.33 ***	0.70 ***	1.00	-									
6. PA-T1	−0.33 ***	−0.15 ***	−0.20 ***	0.57 ***	0.64 ***	1.00	-								
7. GPYDQ-T1	−0.45 ***	−0.17 ***	−0.29 ***	0.77 ***	0.72 ***	0.69 ***	1.00	-							
8. CBC-T2	−0.13 ***	−0.32 ***	−0.14 ***	0.25 ***	0.21 ***	0.18 ***	0.24 ***	1.00	-						
9. PI-T2	−0.17 ***	−0.40 ***	−0.18 ***	0.21 ***	0.24 ***	0.18 ***	0.22 ***	0.74 ***	1.00	-					
10. PA-T2	−0.13 ***	−0.30 **	−0.12 ***	0.18 ***	0.18 ***	0.21 ***	0.21 ***	0.60 ***	0.65 **	1.00	-				
11. GPYDQ-T2	−0.17 ***	−0.41 ***	−0.18 ***	0.22 ***	0.23 ***	0.21 ***	0.27 ***	0.78 ***	0.74 ***	0.69 ***	1.00	-			
12. CBC-T3	−0.23 ***	−0.12 ***	−0.44 ***	0.38 ***	0.40 ***	0.30 ***	0.39 ***	0.20 ***	0.20 ***	0.16 ***	0.23 ***	1.00	-		
13. PI-T3	−0.28 ***	−0.17 ***	−0.54 ***	0.34 ***	0.43 ***	0.29 ***	0.37 ***	0.21 ***	0.26 ***	0.17 ***	0.24 ***	0.78 ***	1.00	-	
14. PA-T3	−0.24 ***	−0.14 ***	−0.42 ***	0.30 ***	0.37 ***	0.33 ***	0.38 ***	0.16 ***	0.18 ***	0.21 ***	0.22 ***	0.72 ***	0.73 ***	1.00	-
15. GPYDQ-T3	−0.29 ***	−0.16 ***	−0.51 ***	0.36 ***	0.42 ***	0.33 ***	0.44 ***	0.20 ***	0.24 ***	0.21 ***	0.26 ***	0.88 ***	0.81 ***	0.83 ***	1.00

*Note*. The controlled variables were gender and age. T1, Time 1 (first year); T2, Time 2 (second year); T3, Time 3 (third year). CESD = Center for Epidemiologic Studies Depression Scale; CBC = cognitive-behavioral competencies; GPYDQ = general positive youth development qualities; PI = positive identity; PA = prosocial attributes. ** *p* < 0.01; *** *p* < 0.001.

**Table 5:** Fit indices of models.

								90% CI for RMSEA	
Model	*χ* ^2^	*df*	CFI	SRMR	AIC	BIC	RMSEA	Low	UP
baseline model	1453.87	112	0.90	0.12	54,276.85	54,559.00	0.10	0.09	0.10
unidirectional model A	987.98	106	0.93	0.11	54,822.95	541,27.02	0.08	0.08	0.09
unidirectional model B	964.16	106	0.94	0.07	53,799.13	53,915.79	0.08	0.075	0.084
bidirectional model	1014.02	100	0.93	0.06	53,863.00	54,203.15	0.085	0.08	0.09
modified model	821.20	98	0.95	0.04	53,672.17	54,017.48	0.08	0.07	0.08

*Note*. All χ2 values are significant at *p* < 0.001. Unidirectional model A = unidirectional model of paths from positive youth development to depressive symptoms; Unidirectional model B = unidirectional model of paths from depressive symptoms to positive youth development.
